# Are generalists more innovative than specialists? A comparison of innovative abilities in two wild sympatric mouse lemur species

**DOI:** 10.1098/rsos.180480

**Published:** 2018-08-15

**Authors:** Johanna Henke-von der Malsburg, Claudia Fichtel

**Affiliations:** 1 Behavioural Ecology and Sociobiology Unit, German Primate Center, Leibniz Institute for Primatology, Kellnerweg 4, 37077 Göttingen, Germany; 2 Department of Sociobiology/Anthropology, Johann-Friedrich-Blumenbach Institute for Zoology and Anthropology, University of Göttingen, Kellnerweg 6, 37077 Göttingen, Germany; 3 Leibniz ScienceCampus ‘Primate Cognition’, Göttingen, Germany

**Keywords:** *Microcebus*, innovative problem-solving, specialist, generalist, personality, inhibitory control

## Abstract

The propensity to flexibly innovate behavioural variants might advantage animals when dealing with novel or modified ecological or social challenges. Interspecific innovative abilities can be predicted by the degree of ecological generalism and intraspecific variation is predicted by personality traits. To examine the effects of these factors on innovation, we compared problem-solving abilities in the generalist grey mouse lemurs (*Microcebus murinus*) and the more specialized Madame Berthe's mouse lemurs (*Microcebus berthae*) in western Madagascar. We examined personality traits by testing 54 individuals in open field and novel object tests, and we assessed problem-solving abilities by presenting an artificial feeding-box that could be opened by three different techniques. The first two techniques presented novel problems and the third technique a modified problem to the more complex second novel problem. In both species, motivation, early success and better inhibitory control characterized innovators and predicted superior problem-solving performance. Although both species performed equally well in finding a solution to the novel problems, the specialist species was more efficient in finding a novel solution to a familiar problem. Since the ecological specialist also exhibited more inhibitory control in this task than the generalist, we propose that specialists may dispose of more efficient problem-solving behaviour.

## 1. Introduction

Changing environmental conditions force animals to find new ways to cope with emerging problems in their natural habitat [1–3]. As resources or niches change dynamically, learning a novel behaviour or modifying an existing behaviour can be advantageous to exploit new resources, or to invade or to create new niches. Hence, the ability to flexibly modify or create novel behavioural variants can impact an individual's fitness [4–6]. One form of behavioural flexibility is innovativeness [7], i.e. the propensity to solve a novel problem or to find a novel solution to an old problem [8]. In particular, foraging innovations are thought to be associated with increased cognitive skills: through associative learning processes, newly discovered motor actions can enhance the expression of acquired foraging behaviours [9]. Learned and innovative behaviour can, hence, be integrated into an individual's behavioural repertoire and can spread through a population (or can be adopted by a population) via social learning [10–12]. Learning is, therefore, essential for both the acquisition and subsequent spread of innovations [13].

To date, research on innovative problem-solving has concentrated mainly on birds and primates providing evidence not only for intraspecific differences in innovative propensity, but also evidence for interspecific variation [14,15]. At the individual level, the propensity to innovate can be influenced by several external and internal factors. For instance, innovation rate can be increased at times of food scarcity when animals need to exploit different food resources or at times of reduced predation risk when animals can afford to be less vigilant while developing new behaviours [4]. By contrast, dietary or time constraints [14,16], as well as exposure to danger, can decrease innovation rate [4,5]. Furthermore, individual characteristics such as age, sex and social rank can affect innovativeness, with adult subordinate males being most innovative (several primate species [7], callitrichid monkeys [17], meerkats [5], birds and primates; reviewed in [18]). For instance, in meerkats (*Suricata suricatta*), subordinate males were most successful in opening an artificial feeding-box [5]. Across primates, innovations were more often reported for males and adults compared to females and younger primates [7].

Additionally, personality is an important factor influencing problem-solving abilities in many species (reviewed in [19], birds [20–24], carnivores [25] and primates [26,27]). Innovators were less neophobic, more motivated or more persistent, compared to less innovative conspecifics [27–30]. For example, in red fronted lemurs (*Eulemur rufifrons*), more persistent individuals were able to solve the most difficult task in a three-task problem-solving experiment [27]. Moreover, behavioural syndromes, i.e. several behavioural outputs that are correlated across various contexts, such as the shy–bold continuum or the proactive–reactive axis [31], differ not only among individuals within species, but also between species. The shy–bold continuum functionally refers mainly to antipredator behaviour and feeding activity where shy individuals would be less likely to approach novel situations, might be less easily trapped and habituate slower to laboratory conditions than bold individuals [31,32]. The proactive–reactive axis is primarily exhibited in exploratory behaviour, fearfulness, aggression and in responses to environmental change. Proactive individuals would control and manipulate environments, whereas reactive individuals would rather respond passively to changing environments. For example, squirrel monkeys (*Samiri sciureus*) were more proactive by being less neophobic and more motivated to engage with apparatuses of a test battery consisting of four problem-solving tasks and showing more rapid and impulsive manipulation techniques than titi monkeys (*Callicebus moloch*) [33]. Titi monkeys, by contrast, were more reactive by being more neophobic, manipulating the apparatuses more slowly and tentatively. Moreover, this difference in being more pro- or reactive has been suggested to be associated with ecological generalism: the more proactive squirrel monkeys are ecological generalists, whereas titi monkeys are ecologically more specialized [33].

At the species level, ecological generalism has also been suggested to influence innovativeness [34–36]. Ecological generalists inhabit a broad ecological niche which exposes them to diverse environmental conditions and many potential food resources. Hence, they depend on explorative behaviour to adapt to the variety of environmental circumstances [34] as well as on explorative foraging techniques to benefit from the variety of the locally and or seasonally available food [37]. Specialists differ from generalists in niche width within an ecological gradient [38]. While stable environmental conditions favour specialists, as they are using resources more efficiently than generalists [38], unpredictable or complex environments favour generalists that do not rely on a few specific, but several resources [38,39]. Ecological generalism, as well as patchily distributed food, extractive foraging and flexible responses to fluctuating environments have been suggested to be associated with superior cognitive abilities in birds and primates [40]. Moreover, in comparison to specialist species, generalists are, on average, less neophobic, supposedly because they encounter more habitats or food types early in life [39]. Furthermore, low neophobia and high exploration appear to be correlated with the propensity to innovate [39,41,42]. Accordingly, in insects, birds and primates, generalist species are also more explorative and exhibited higher innovation rates than specialists [3,29,43].

Despite these interspecific differences in problem-solving performance, a direct comparison of generalist and specialist species regarding the influence of personality traits on the propensity to innovate has not yet been conducted. In the present study, we examined the influence of individual characteristics on innovative problem-solving abilities in a generalist and a specialist species of Malagasy primates. The study of cognition of the most basal living primates, the Malagasy lemurs, is specifically interesting, as they retain behavioural and cognitive traits that characterize most living monkeys and apes (haplorrhines) [44]. Recent studies using a comprehensive test battery on cognitive abilities in the physical and social domain, i.e. the Primate Cognition Test Battery [45], revealed that the average performance of lemurs was not different from that of the haplorrhines in many aspects. Specifically, lemurs' overall performance was slightly inferior in the physical domain, but matched that of haplorrhines in the social domain [46,47].

Among lemurs, mouse lemurs are an excellent model species for the present study, because this genus consists of sympatric species that exhibit variation in habitat and food specialization. Personality traits and cognitive abilities have already been studied in the grey mouse lemur (*Microcebus murinus*) in captivity and the wild [48–59]. This species is an ecological generalist inhabiting various forest types, from evergreen littoral rainforests in southern Madagascar to seasonal dry deciduous forests in western and northwestern Madagascar [60]. By contrast, the Madame Berthe's mouse lemur (*Microcebus berthae*) is a habitat specialist, occurring only in the Menabe Central region of central western Madagascar [61]. In Kirindy Forest, the two species occur in sympatry [62–64]: Madame Berthe's mouse lemurs’ home ranges (females: 2.5 ha, males: 4.9 ha) are twice the size of those of grey mouse lemurs (females: 1.8 ha, males: 3.2 ha), and both species show high interindividual home-range overlap [65].

Although the two species feed on the same food sources and show high feeding overlap, they differ in the proportions of ingested food categories, reflecting different degrees of dietary specialization [40,63,64,66]. Madame Berthe's mouse lemurs feed mainly (82%) on sugary secretions produced by the homopteran larvae of the flower bug (*Flatida coccinea*) and further supplement their diet by animal matter (11.4%) according to the seasonal availability of arthropods. They also feed occasionally on fruits and flowers (2%) and gum (0.2%) but much less often than grey mouse lemurs (homopteran secretions (59.5%); animal matter (16.6%), fruits and flowers (8.6%) and gum (9.2%)) [64]. As Madame Berthe's mouse lemurs exhibit the narrowest feeding niche known for lemurs (annual mean niche breadth: 0.12, based on Levin's standardized index), they are limited in their flexible choice of food sources, especially during periods of reduced food availability. Grey mouse lemurs instead exhibit a larger dietary breadth (annual mean: 0.62) and adapt their dietary composition more to seasonal availability [63,64].

Grey mouse lemurs exhibit several personality traits, as shown in captivity [48] and in the wild [49,50,64]. Captive grey mouse lemurs exhibit behavioural consistency along a shy–bold axis with shyer individuals being less aggressive and more neophobic than bolder individuals [48]. Wild grey mouse lemurs also exhibit consistent individual differences in activity, exploration and boldness [50]. In addition, boldness correlates with risk-taking behaviour with bolder individuals being more likely to feed on high-risk platforms placed on the ground compared to low-risk platforms placed at a height of 1.5 m [49]. As mouse lemurs only occasionally feed on the ground, feeding platforms on the ground present a higher predation risk than a feeding platform situated at a height within the usual foraging range of 1–6 m [64]. Moreover, grey mouse lemurs have already been subject to various studies on cognition in captivity on discriminative abilities [51,55], spatial memory [53,55,67], problem-solving performance [56] and in the wild on spatial memory and problem-solving abilities [57,68,69]. For example, captive and wild mouse lemurs are able to learn specific motor patterns to manipulate various artificial problem-solving apparatuses [56,69]. However, the present study is the first assessing personality traits and cognitive abilities in Madame Berthe's mouse lemurs.

As innovations in the wild happen rarely and require long-term studies of the behavioural repertoire of a population [5,19], we examined innovative problem-solving abilities in mouse lemurs experimentally, as has already been done in other species (primates [17,27,28,70], carnivores [5,71] and birds [43,72–74]). We confronted the two species of mouse lemurs with three extractive foraging tasks with increasing complexity of the opening mechanism to access a food reward inside of a problem-solving box. To solve the first two tasks, study subjects had to find a solution to a novel problem, referring to the first part of the definition for innovation (i.e. to find a solution to a novel problem), whereas the third, most complex task referred to the second part of the definition for innovation (i.e. to modify the solution of an old problem).

In addition, we examined how personality traits influence individual innovative tendencies in mouse lemurs by conducting open field and novel object tests [49,50] to measure an individual's propensity to explore an open unknown area [75] as well as its neophilia and exploration towards unknown objects [76]. We predicted that (i) more explorative and neophilic, but also more motivated, more persistent and less conservative individuals should show superior innovative abilities. In addition, we predicted that (ii) grey mouse lemurs are more explorative and more neophilic than Madame Berthe's mouse lemurs due to their greater level of ecological generalism. Finally, as interspecific comparisons on innovative abilities suggest that success in problem-solving is associated with foraging behaviour and habitat use [33,34,37,44], we also predicted that (iii) grey mouse lemurs should be more successful in problem-solving tasks than Madame Berthe's mouse lemurs.

Finally, we addressed possible motivational confounds which are likely in food-rewarded tasks, especially when testing wild animals at times of food shortage, by measuring the individuals' current body condition via a body mass index (BMI) [77]. We used this measure as proxy for the individuals’ energetic state and, hence, their feeding motivation, as we could not control for food intake prior to testing, as would be possible with captive animals.

## 2. Material and methods

### 2.1. Study site

We conducted this study in Kirindy Forest (mean annual temperature: 24.7°C, annual precipitation: 800 mm), which is approximately 60 km northeast of Morondava in central western Madagascar. The dense dry deciduous forest is located within a 12 500 ha forest concession operated by the Centre National de Formation, d'Etudes et de Recherche en Environnement et Foresterie (CNFEREF) of Morondava. This site is characterized by pronounced seasonality with a dry season from May to October and a hot rainy season from November to April [78]. The study area, characterized by a relatively high density of trees, dead wood and lianas [64], is equipped with a rectangular grid system of small trails in 25 m intervals within a 500 × 500 m^2^ core area, and each trail intersection is marked by a letter–number code for orientation [79].

Data collection took place at the end of the dry season when food availability was low and predation risk high [78]. Mouse lemurs undergo seasonal reproduction with the mating season starting in October for grey mouse lemurs and in November for Madame Berthe's mouse lemurs [80]. As female grey mouse lemurs hibernate during the dry season, they are in relatively poor body condition before entering the mating season, whereas male grey mouse lemurs and Madame Berthe's mouse lemurs are in better body condition at that time of the year as they stay active or only enter short-term torpor during the dry season [81,82]. Energy expenditure might have been different between the two species at that time, as Madame Berthe's mouse lemurs, although being in better body condition compared to grey mouse lemurs, were extremely restricted in their choice of food sources, by being nearly completely limited to homopteran secretions. Grey mouse lemurs instead could still vary their diet and, specifically, feed on other stable resources like gum.

### 2.2. Capture and housing

At dusk, we set up to 96 Sherman live traps (7.5 × 9.0 × 23.5 cm^3^) baited with a slice of banana at a height of 0.5–2.0 m at the trail intersections of an area where both species occur in sympatry [83]. We controlled and closed the traps at dawn and brought captured mouse lemurs to the nearby research station, where we weighed newly captured individuals, restrained them briefly with 0.02–0.04 ml of ketamine (50 mg ml^−1^) to take standard morphometric measurements and marked them individually with subdermal implanted microtransponders (Trovan, Usling, Germany). Recaptured individuals were only controlled for their identity and weighed. We calculated the BMI as the proportion of body mass (in g) and body length (in mm).

To conduct the experiments, mouse lemurs were housed in a closed room to protect them from predators and to ensure an undisturbed testing environment. They were housed individually in a cage of about 80 × 80 × 80 cm^3^ equipped with a nesting box, branches for climbing and an experimental platform. Animals were fed with banana and insects (cockroaches, moths or locusts) each night after conducting the experiments and had ad libitum access to water. Animals were released at dusk at their site of capture after a maximum of three (*N* = 59 cases) or four (*N* = 33 cases) nights. In total, we tested 36 grey mouse lemurs (11 females and 25 males) and 18 Madame Berthe's mouse lemurs (nine females and nine males) that have mostly been captured for the first time, supposing a median age of under 1 year (grey mouse lemurs: 0–2 years, Madame Berthe's mouse lemurs: 0–1 years) as regular monthly captures are conducted for 15 years in Kirindy Forest. Testing took place each night from 19.00 to 2.00 latest, depending on the number of individuals being tested in that night and their motivation to participate. The individuals' testing order differed randomly within each of the three or four consecutive nights.

### 2.3. Open field and novel object test

**Figure 1.**
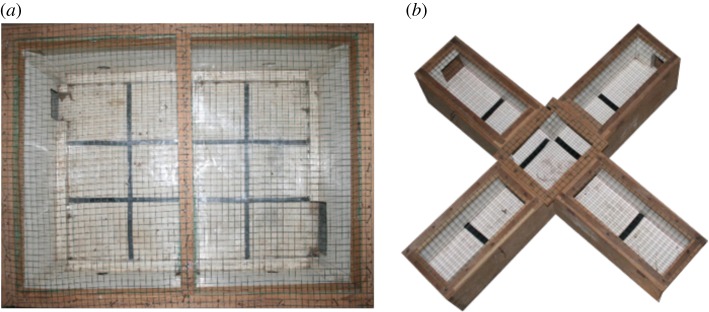
Open fields: (*a*) wooden box (80 × 60 × 60 cm^3^) and (*b*) maze (each arm: 40 × 16 × 15 cm^3^).

We conducted open field tests to examine personality variations in activity and exploration in novel environments [76,84]. We used two different open fields to control for repeatability of this personality trait. The first open field test was conducted in the first night before the subject was released into the experimental cage, whereas the second open field test was conducted in the last night after the animal had finished the three innovation tasks. The two open fields consisted either of a wooden box of about 80 × 60 × 60 cm^3^ with a plotted grid of 12 uniform cells (20 × 20 cm^2^), four blind holes and two bigger entrances (figure 1*a*), or of a wooden maze with a plotted grid of nine uniform cells (16 × 20 cm^2^) and four arms, each of about 40 × 16 × 15 cm^3^, and with one closed entrance (figure 1*b*). The order of the two types of open fields differed randomly between subjects.

A test session lasted 5 min, starting with the subject being introduced into the arena, either into one corner of the box or the centre of the maze. All experiments were conducted under dimmed red-light conditions and were video-taped with a digital camcorder (Sony HDR-CX 240). Based on these video-recordings, we measured the subjects’ latency to their first movement, the duration spent locomoting, being vigilant and sniffing, the number of jumps as well as the number of grid cells traversed (table 1) to assess personality variation in active and passive exploration in novel environments [50]. By dividing the number of grid cells a subject traversed by the duration it spent locomoting, we calculated the speed with which the subject explored the open field. Since the grid cell sizes in the two open fields differed slightly, the absolute exploration speeds can be directly compared only with caution.

**Table 1.** Combined factors of variables extracted from the open field and novel object tests with descriptions and respective ICC.

variable	description	ICC
*active exploration*		−0.057
latency first movement	latency from the introduction of the subject into the open field to its first movement	
locomoting	duration of body movements with at least the two forefeet	
jump rate	number of jumps between the bottom and the top	
number of grid cells	number of grid cells traversed	
*passive exploration*		−0.244
watching	duration of only head movements of at least 90°	
sniffing	duration of touching the wall with the nose	
*neophilia*		0.179
latency first contact	latency from the introduction of the novel object to the subject's first contact with it	
contact	duration spent in contact with the novel object	
*exploration speed*	number of grid cells traversed per time spent locomoting	−0.434

**Figure 2.**
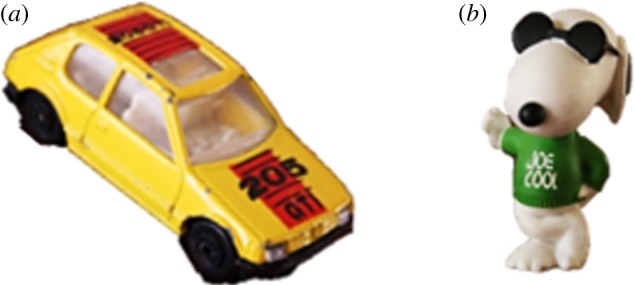
Examples for novel objects: (*a*) toy car and (*b*) Snoopy.

Directly after the open field test, we conducted a 5 min novel object test by depositing a novel object either in the opposite corner of the box, depending on the subject's momentary position, or in the middle of the maze. By confronting the subjects with novel objects, we measured their neophilia and exploration towards unknown objects without food rewards [76]. As novel objects, we used either one of five metallic toy cars (7.0–7.5 cm in length; figure 2*a*) or one of five plastic ‘Snoopys’ (5.0–5.2 cm in height; figure 2*b*). The objects differed randomly between subjects. For the second novel object test, we used the respective other object type (car or Snoopy) as in the first test. We measured subjects' latency to approach the object and the duration spent in contact with it (table 1). Before each experiment, we cleaned both the open fields and the novel objects, with 80% ethanol to remove potential olfactory cues left by previous subjects that might have influenced the subjects’ behaviour.

### 2.4. Problem-solving experiment

**Figure 3.**
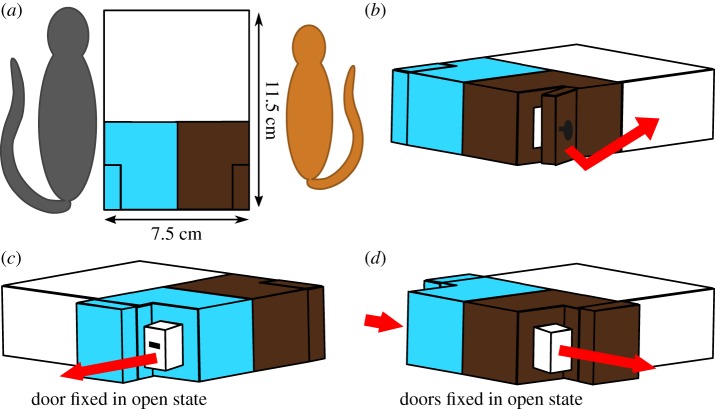
Novel problem-solving box. Arrows indicate the direction of the opening mechanism. (*a*) Closed box and its dimensions in relation to grey mouse lemurs (grey) and Madame Berthe's mouse lemurs (orange). (*b*) Task 1: slightly opened hinged door. (*c*) Task 2: drawer slightly pulled out of the box. (*d*) Task 3: drawer slightly pushed out of the box.

After the first open field and novel object test, animals were released into the experimental cage, in which we conducted the novel problem-solving experiment during the subsequent three nights. We used an artificial wooden box (11.5 × 7.5 × 3.0 cm^3^), which consisted of two hinged doors, one on each side, that could be fixed via screws in an open or closed state (figure 3*a*). Additionally, a drawer with a handle on one side could be placed inside the box. Each side was painted in a different colour (dark brown and light blue) to provide a visual cue for the two sides. Testing order differed randomly between the individuals depending on the number of individuals being tested within the same night (*N*_min_ = 3, *N*_max_ = 7, median = 6 individuals) and their motivation to participate. All experiments were conducted under dimmed red-light conditions and were video-taped with a digital camcorder (Sony HDR-CX 240).

Prior to the actual experiment, we placed the box with both doors open, without the drawer, and equipped with a small piece of banana on the experimental platform inside the subject's cage to familiarize them with the problem-solving box. Subjects had to retrieve the piece of banana six times on each side of the box to ensure that they had learned to retrieve the reward from both sides of the box. When the subject did not move for 20 min and did not approach the box, we stopped the trial and repeated it later or during the following night. Only if the subject retrieved the piece of banana six times on both sides of the box did we start with the actual problem-solving experiment.

The problem-solving experiment consisted of three tasks with increasing complexity to obtain access to a food reward. In the first novel problem task, only the hinged door on one side of the box had to be opened (figure 3*b*). In the second novel problem task, the previous opening mechanism, the hinged door on one side of the box was blocked, and the subject had to explore the other side, where the door was fixed in the open state and the drawer containing the reward was placed inside the box with the handle facing the open door. To access the reward, subjects had to pull the drawer out of the box (figure 3*c*). In the third task, both hinged doors were fixed in the open state and the drawer was placed inside the box with the handle facing the same side as in the second task, but the pulling mechanism of the drawer was blocked. Hence, subjects had to first push the drawer out of the box and then move to the other side to retrieve the reward (figure 3*d*). This third task represented a modified problem.

Before each session, we cleaned the box with 80% ethanol to exclude olfactory cues left by previous subjects that might have influenced the next subject's behaviour. For each trial, the box was baited out of the subject's sight with a small piece of banana. A trial started when the box was placed on the experimental platform and lasted for 5 min. Only in the very first trial of the first task, the trials lasted for 10 min to give the subjects more time to familiarize themselves with the new problem. A session consisted of 12 trials but was stopped earlier if the subject did not inspect the box in two trials. If the subject solved the task in at least 10 trials within a given session, the respective opening mechanism was considered as learned and the subsequent task was started in the following session. We conducted one to five sessions (median = 2) per subject and night, depending on the subject's motivation.

If the subject did not solve the task twice in a row, we introduced an intermediate training of either opening the door slightly in the first task (*N* = 91 out of 838 trials ≙ 10.9%), pulling the drawer slightly out of the box in the second task (*N* = 106 out of 663 trials ≙ 16.0%) or pushing the drawer slightly out of the box in the third task (*N* = 40 out of 465 trials ≙ 8.6%). If the subject also did not solve the task within this help trial, we increased the given help by opening the door or pulling the drawer a bit more out of the box as in the help trial before. If the subject did not solve the task in the subsequent two normal non-help trials after a solved help trial, we repeated the help trial. If the subject remained unsuccessful without help, we repeated this procedure until the end of the session. When a help trial was solved, we proceeded with the normal testing in the subsequent trial. This intermediate training was basically needed for individuals that seemed to be less persistent or motivated to explore the box sufficiently in order to find the opening mechanism themselves. However, we did not include help trials in the final analyses because the two species did not differ in their number of help trials (general linear mixed effects models (GLMMs) (binomial response: ‘help’ (0: no help given, 1: help given); fixed factors: task, species; random effect: ID; random slope: task); comparison with null model (excl. fixed factors): *p* = 0.472; individual effects (comparison with respective reduced model): task: *p* = 0.285, species: *p* = 0.938).

### 2.5. Video analyses

Video-recordings were analysed at half speed by using Windows Movie Maker v. 16.4.3528.0331 (©2012 Microsoft Corporation). We recorded the time until success (i.e. retrieving the reward), the number of successful and unsuccessful trials, and calculated the success rate as the proportion of successful trials to total trials, excluding errors.

We counted the number of unsuccessful trials until the first successful trial as the number of errors. Furthermore, we measured the latency to approach, i.e. the time from the beginning of the trial until the subject approached the task, the time it was in contact with it and the time it spent actively manipulating the box. Persistence was calculated as the ratio of the time actively manipulating the task to the time being in contact with it. To measure whether inhibitory control influenced problem-solving abilities, we measured the change latency as the time an individual needed to abandon the previously successful opening technique. By definition, the change latency could only be extracted for Tasks 2 and 3 (table 2). For subsequent statistical analyses, we used the arithmetic means of the success latency, approach latency, persistence and change latency per individual and task.

**Table 2.** Evaluated variables for the novel problem-solving experiment and respective descriptions.

variable	description
approach latency	time until the first orientation (facing the box with both eyes) on the platform or the first contact with the box
persistence	ratio of time manipulating by mouth or hands to total time in contact with the box (any kind of box contact, including sniffing)
change latency	time until the previously successful technique was no longer used (n.a. for Task 1)
errors	number of failed trials until the first successful trial
success latency	time until success, subtracted by approach latency (n.a. for not solved trials)
success rate	rate of solved trials to total trials (excluding errors)
solved	solved task criterion: success in at least 10 out of 12 trials during a given session

A second person naive to the research question analysed 10% of the videos a second time to assess inter-rater reliability, which was 90.5% for the open field and novel object tests and 99.3% for the problem-solving experiment (intraclass correlation coefficient; R package ‘ICC’ [85]).

### 2.6. Statistical analyses

We performed all statistical analyses with R v. 3.4.1 (©2017, The R Foundation for Statistical Computing). We combined the variables measured in the open field and novel object tests according to Dammhahn [50] into four factors (active exploration, passive exploration, neophilia and speed; table 1). By using a principal component analysis, we retained the loadings of the first principal component of each factor. To identify repeatable personality traits, we calculated the intraclass correlation coefficient (R package ‘ICC’ v. 2.3.0 [85]) between factors extracted from the first and from the second open field and novel object tests of individuals which did both tests twice (*N* = 17).

Only the factor ‘exploration speed’ (grid cells traversed per time spent locomoting) was repeatable (ICC = −0.434) and, thus, used as a personality trait for further analyses. The non-repeatable factors such as active exploration (ICC = −0.057), passive exploration (ICC = −0.244) and neophilia (ICC = 0.179; table 1) were not considered as personality traits as they are defined as being consistent across varying contexts and times [86]. We also examined whether the latency to approach, the persistence and the change latency were repeatable over the problem-solving tasks as they could reflect personality differences between individuals.

We used a Mann–Whitney *U* test to test for interspecific differences in the four factors derived from the first open field and novel object tests and to test whether individuals which participated in the problem-solving experiment differed from ‘non-participators’ in these factors. Differences between the proportion of subjects which participated and finished the problem-solving tasks were analysed with a Fisher's exact test.

To evaluate problem-solving abilities, we used three performance measures: (i) the probability to solve the problems (task solved/not solved), (ii) the success latency, and (iii) the success rate (number of successes/failures, excluding errors). We used binomial GLMMs (R function glmer, R package ‘lme4’ v. 1.1–13 [87]) to examine factors influencing the probability to solve the problems (task solved = 1 and task not solved = 0) and success rate and linear mixed effects models (LMMs; R function lmer, R package ‘lme4’ v. 1.1–13 [87]) to examine factors influencing the success latency.

Since we were restricted to use less complex linear models due to the relatively small sample size in Task 3, we split the analyses of influences on the three performance measures into the following steps. First, we examined whether species or task or their interaction influenced the respective performance measure. If their interaction was significant, we used this interaction term rather than the single terms as control for species and task for all following models. Second, we examined whether the personality trait exploration speed (*z*-transformed to a mean of 0 and a standard deviation of 1) or BMI (*z*-transformed to a mean of 0 and a standard deviation of 1) or their interactions with species and/or task influenced the performance measures. If so, we controlled for these variables and interactions in addition to species and task in the following models. If not, we kept on controlling only for species and task or their interaction, respectively. Finally, we examined the influence of each variable of the problem-solving experiment (approach latency, persistence, errors (each *z*-transformed to a mean of 0 and a standard deviation of 1) and change latency (log-transformed and *z*-transformed to a mean of 0 and a standard deviation of 1); table 2) separately, also checking for interactions with species and/or task. In all models, we included individual ID as a random factor.

We did not include sex as a fixed factor in any of the models because only six females per species participated in the experiments. Where applicable, we controlled for normal residual distribution, homoscedasticity and collinearity, as well as for model stability and variance inflation factors (R function vif, R package ‘car’ [88]). Significance testing for the individual effects was based on likelihood ratio tests comparing the full with respective reduced models [89] (R function drop1).

## 3. Results

### 3.1. Personality traits

Of the personality factors derived from the open field and novel object tests, only exploration speed was repeatable (ICC = −0.434), whereas active (ICC = −0.057) and passive exploration (ICC = −0.244) as well as neophilia (ICC = 0.179) were not (table 1). Of the personality factors derived from the problem-solving tasks, latency to approach (ICC = 0.632), persistence (ICC = 0.416) and the change latency (ICC = −0.316) were repeatable. The two species did not differ in their active exploration (*U* = 1077, *p* = 0.073), exploration speed (*U* = 687, *p* = 0.097) or neophilia (*U* = 917, *p* = 0.706) during the first open field and novel object tests. There was a trend for grey mouse lemurs to spend less time passively exploring the open field (*U* = 1089, *p* = 0.057).

### 3.2. Participation

Twelve grey mouse lemurs and six Madame Berthe's mouse lemurs did not familiarize with the artificial box. Twenty-four grey mouse lemurs (six females and 18 males) and 12 Madame Berthe's mouse lemurs (six females and six males) participated in the problem-solving experiment. The proportions of individuals that participated in the three tasks did not differ between species (Fisher's exact test: Task 1: *N*_Mbe_ = 12 out of 18, *N*_Mmu_ = 24 out of 36, *p* = 1; Task 2: *N*_Mbe_ = 11 out of 12, *N*_Mmu_ = 17 out of 18, *p* = 1; Task 3: *N*_Mbe­_ = 10 out of 11, *N­*_Mmu_ = 12 out of 17, *p* = 0.355; Mmu: grey mouse lemurs, Mbe: Madame Berthe's mouse lemurs; table 3). Individuals that familiarized themselves with the novel problem box and subsequently participated in the innovation experiment spent more time passively exploring the open field test than those that did not familiarize themselves with the novel problem box (Mann–Whitney *U* test: *U* = 402, *p* = 0.044). However, participating individuals did not differ in active exploration, exploration speed and neophilia from those that did not participate (Mann–Whitney *U* test: active exploration: *U* = 340, *p* = 0.778; exploration speed: *U* = 429, *p* = 0.055; neophilia: *U* = 312, *p* = 0.832).

**Table 3.** Numbers of individuals per species which participated and solved the respective problem-solving task, as well as the respective percentage of individuals that solved the task.

species (total number)	task	participated	solved	solved per participated (%)
grey mouse lemurs (*N* = 36)	1	23	18	78
	2	17	16	94
	3	12	6	50
Madame Berthe's mouse lemurs (*N* = 18)	1	12	11	92
	2	11	10	91
	3	10	7	70

### 3.3. Problem-solving

Both species were able to open the box with all three opening techniques. Task 1 was solved by 81%, Task 2 by 93% and Task 3 only by 59% of the individuals participating in the respective task (table 3). The probability to solve the tasks did not differ between grey and Madame Berthe's mouse lemurs but between tasks, with Task 2 being solved by more individuals and Task 3 by fewer individuals than Task 1 (table 4*a*). The interaction between species and task was not significant (*p* = 0.632) and, thus, not included in the following models. Since exploration speed and BMI did not influence the probability to solve the tasks (table 4*b*), they were also excluded from the following models. Individuals which solved the tasks approached the box earlier (table 4*c*), manipulated more persistently (table 4*d*), made fewer errors (table 4*e*) and abandoned the previously successful manipulation technique earlier (table 4*f*) than those which did not solve the tasks.

**Table 4.** Statistics for binomial GLMMs testing effects of (*a*) species and task, (*b*) exploration speed and BMI, (*c*) approach latency, (*d*) persistence, (*e*) errors prior to first success and (*f*) inhibitory control (change latency) on the propensity to solve the tasks (solved = 0, not solved = 1). Individual ID was always included as a random effect. The first *p*-value is extracted from the summary of the respective model, whereas the second *p*-value is extracted from the model comparison with the respective reduced model (function drop 1). The test statistics show the results of the comparison of the full to the null model.

	estimate	s.e.	*p*-value	*p*-value
**(*a*) fixed factors**				
intercept (Mme Berthe's mouse lemurs, Task 1)	2.000	1.201	0.096	
grey mouse lemurs	−0.792	0.799	0.322	0.194
Task 2	1.113	0.876	0.204	0.010
Task 3	−1.190	1.254	0.343	
*test statistics*	*χ*² = 10.285	d.f. = 3	*p* = 0.016	
**(*b*) fixed factors**				
intercept (Mme Berthe's mouse lemurs, Task 1)	2.207	1.320	0.094	
grey mouse lemurs	−0.118	1.401	0.425	0.398
Task 2	1.101	0.870	0.206	0.010
Task 3	−1.202	1.125	0.286	
speed^a^	−0.055	0.281	0.844	0.844
BMI^a^	0.190	0.646	0.768	0.768
*test statistics*	*χ*² = 10.408	d.f. = 7	*p* = 0.167	
**(*c*) fixed factors**				
intercept (Mme Berthe's mouse lemurs, Task 1)	3.388	0.941	<0.001	
grey mouse lemurs	−1.388	0.808	0.086	0.063
Task 2	0.244	0.992	0.806	0.001
Task 3	−2.465	0.832	0.003	
approach latency^a^	−1.624	0.488	0.001	<0.001
*test statistics*	*χ*² = 29.923	d.f. = 4	*p* = <0.001	
**(*d*) fixed factors**				
intercept (Mme Berthe's mouse lemurs, Task 1)	5.324	1.393	<0.001	
grey mouse lemurs	−1.363	0.850	0.109	0.091
Task 2	−1.391	1.177	0.237	<0.001
Task 3	−5.069	1.487	0.001	
persistence^a^	2.744	0.706	<0.001	<0.001
*test statistics*	*χ*² = 42.262	d.f. = 4	*p* = <0.001	
**(*e*) fixed factors**				
intercept (Mme Berthe's mouse lemurs, Task 1)	2.347	0.715	0.001	
grey mouse lemurs	−1.232	0.702	0.079	0.062
Task 2	1.455	0.930	0.118	0.003
Task 3	−1.326	0.658	0.044	
errors^a^	−0.713	0.308	0.020	0.019
*test statistics*	*χ*² = 15.803	d.f. = 4	*p* = 0.003	
**(*f*) fixed factors**				
intercept (Mme Berthe's mouse lemurs, Task 1)	4.115	1.734	0.018	
grey mouse lemurs	−1.710	1.577	0.278	0.220
Task 3	1.503	1.433	0.294	0.279
change latency^a,b^	−3.013	1.171	0.010	0.002
*test statistics*	*χ*² = 14.204	d.f. = 3	*p* = 0.003	

^a^*z*-transformed to a mean of 1 and a standard deviation of 0.

^b^Log-transformed.

### 3.4. Problem-solving performance: success latency

**Figure 4.**
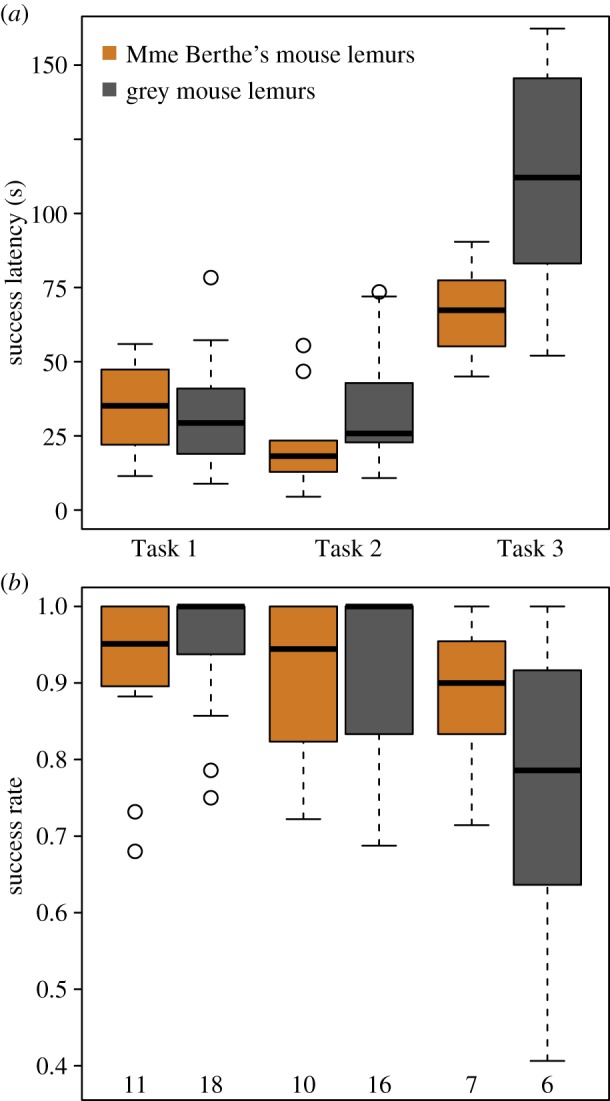
Problem-solving performance as success latency (*a*) and success rate (*b*) in two novel problems (Tasks 1 and 2) and one modified problem (Task 3) of two species of mouse lemurs. Shown are median, interquartile range, minimum–maximum range and outliers. Number of individuals as indicated below each boxplot. (*a*) The success latency was longer in Task 3 than in Tasks 1 and 2. Species differences were not significant, as was the interaction between species and task. (*b*) The success rate was lower in Task 3 than in Tasks 1 and 2. Species difference was significant for Task 3, with Madame Berthe's mouse lemurs having a greater success rate than grey mouse lemurs.

There was a trend for the two species to differ in their success latency, with grey mouse lemurs having tentatively longer success latencies than Madame Berthe's mouse lemurs. Task had a significant influence on success latency, with the shortest latency in Task 2 and the longest latency in Task 3 (table 5*a* and figure 4*a*). Since the interaction between species and task was not significant (*p* = 0.077), and exploration speed as well as BMI (table 5*b*) did not influence success latency they were excluded from the following models.

**Table 5.** Statistics for LMMs testing effects of (*a*) species and task, (*b*) exploration speed and BMI, (*c*) approach latency, (*d*) persistence, (*e*) errors prior to first success and (*f*) inhibitory control (change latency) on the success latency. Individual ID was always included as a random effect. The *p*-value is extracted from the model comparison with the respective reduced model (function drop 1). The test statistics show the results of the comparison of the full to the null model.

	estimate	s.e.	*p*-value
**(*a*) fixed factors**			
intercept (Mme Berthe's mouse lemurs, Task 1)	3.199	0.137	
grey mouse lemurs	0.255	0.141	0.074
Task 2	−0.192	0.153	<0.001
Task 3	1.076	0.190	
*test statistics*	*χ*² = 35.392	d.f. = 3	<0.001
**(*b*) fixed factors**			
intercept (Mme Berthe's mouse lemurs, Task 1)	2.934	0.243	
grey mouse lemurs	0.698	0.370	0.063
Task 2	−0.196	0.150	<0.001
Task 3	1.108	0.187	
speed^a^	−0.088	0.068	0.199
BMI^a^	−0.244	0.183	0.225
*test statistics*	*χ*² = 3.260	d.f. = 2	0.196
**(*c*) fixed factors**			
intercept (Mme Berthe's mouse lemurs, Task 1)	3.137	0.135	
grey mouse lemurs	0.266	0.136	0.054
Task 2	−0.091	0.155	<0.001
Task 3	1.168	0.188	
approach latency^a^	0.157	0.070	0.028
*test statistics*	*χ*² = 40.192	d.f. = 4	<0.001
**(*d*) fixed factors**			
intercept (Mme Berthe's mouse lemurs, Task 1)	3.136	0.134	
grey mouse lemurs	0.217	0.132	
Task 2	−0.010	0.166	<0.001
Task 3	1.230	0.189	
persistence^a^	−0.344	0.111	
persistence^a^:Grey mouse lemur	0.281	0.134	0.039
*test statistics*	*χ*² = 44.373	d.f. = 5	<0.001
**(*e*) fixed factors**			
intercept (Mme Berthe's mouse lemurs, Task 1)	3.186	0.139	
grey mouse lemurs	0.277	0.147	0.064
Task 2	−0.0798	0.153	<0.001
Task 3	1.091	0.192	
errors^a^	0.036	0.074	0.622
*test statistics*	*χ*² = 35.636	d.f. = 4	<0.001
**(*f*) fixed factors**			
intercept (Mme Berthe's mouse lemurs, Task 2)	3.168	0.169	
grey mouse lemurs	0.337	0.177	0.064
Task 3	0.646	0.239	0.010
change latency^a,b^	0.404	0.119	0.002
*test statistics*	*χ*² = 45.052	d.f. = 3	<0.001

^a^*z*-transformed to a mean of 1 and a standard deviation of 0.

^b^Log-transformed.

**Figure 5.**
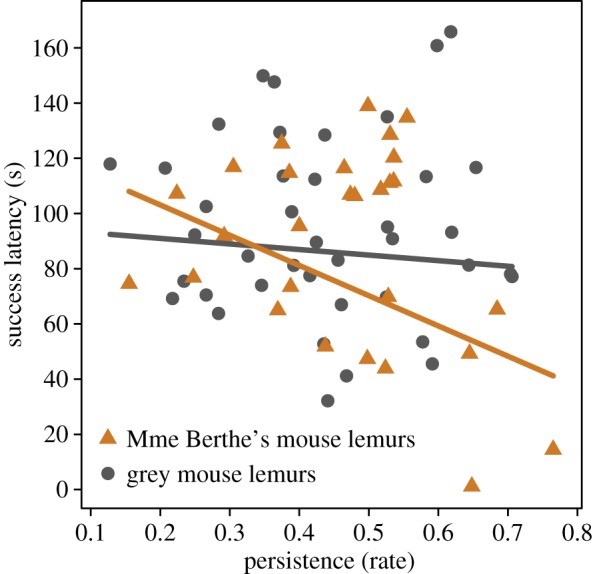
Influence of the persistence (rate of time spent manipulating to total time in contact with the problem-solving box) on the success latency. With greater persistence success latency decreased in both species, showing a more prominent effect for Madame Berthe's mouse lemurs (orange triangles) than for grey mouse lemurs (grey circles).

Individuals that approached the task later had longer success latencies (table 5*c*). Persistence influenced success latency in interaction with species: the more persistent individuals manipulated the task, the faster they succeeded and this effect was more prominent for Madame Berthe's mouse lemurs then for grey mouse lemurs (table 5*d* and figure 5). The number of errors had no effect on success latency (table 5*e*). The earlier the individuals changed their previous technique, the faster they succeeded (table 5*f*).

### 3.5. Problem-solving performance: success rate

The interaction between species and task was significant for the success rate (*p* = 0.001), with grey mouse lemurs having a lower success rate than Madame Berthe's mouse lemurs in Task 3, but not in Tasks 1 and 2 (table 6*a* and figure 4*b*). This interaction term was subsequently included in all following models. Exploration speed and BMI did not influence the success rate (table 6*b*) and were, thus, not included in the following models.

**Table 6.** Statistics for binomial GLMMs testing effects of (*a*) species and task, (*b*) exploration speed and BMI, (*c*) approach latency, (*d*) persistence, (*e*) errors prior to first success and (*f*) inhibitory control (change latency) on the success rate (relation between the number of solved and failed trials). Individual ID was always included as a random effect. The first *p*-value is extracted from the summary of the respective model, whereas the second *p*-value is extracted from the model comparison with the respective reduced model (function drop 1). The test statistics show the results of the comparison of the full to the null model.

	estimate	s.e.	*p*-value	*p*-value
**(*a*) fixed factors**				
intercept (Mme Berthe's mouse lemurs, Task 1)	2.450	0.373	<0.001	
grey mouse lemurs	0.766	0.501	0.126	
Task 2	−0.132	0.355	0.709	
Task 3	−0.349	0.395	0.377	
grey mouse lemurs: Task 2	−0.466	0.513	0.363	0.001
grey mouse lemurs: Task 3	−1.923	0.550	<0.001	
* test statistics*	*χ*² = 42.596	d.f. = 5	*p* ≤ 0.001	
**(*b*) fixed factors**				
intercept (Mme Berthe's mouse lemurs, Task 1)	2.689	0.579	<0.001	
grey mouse lemurs	0.375	0.925	0.686	
Task 2	−0.126	0.356	0.723	
Task 3	−0.416	0.413	0.315	
grey mouse lemurs: Task 2	−0.444	0.513	0.388	0.004
grey mouse lemurs: Task 3	−1.853	0.575	0.001	
speed^a^	0.134	0.233	0.567	0.557
BMI^a^	0.178	0.398	0.656	0.657
*test statistics*	*χ*² = 43.189	d.f. = 7	*p* ≤ 0.001	
**(*c*) fixed factors**				
intercept (Mme Berthe's mouse lemurs, Task 1)	2.947	0.460	<0.001	
grey mouse lemurs	0.451	0.559	0.420	
Task 2	−0.770	0.437	0.078	
Task 3	−1.045	0.481	0.030	
grey mouse lemurs: Task 2	0.046	0.564	0.935	0.016
grey mouse lemurs: Task 3	−1.422	0.597	0.017	
approach latency^a^	−0.455	0.163	0.005	0.004
*test statistics*	*χ*² = 50.949	d.f. = 6	*p* ≤ 0.001	
**(*d*) fixed factors**				
intercept (Mme Berthe's mouse lemurs, Task 1)	2.998	0.403	<0.001	
grey mouse lemurs	0.933	0.497	0.061	
Task 2	−0.821	0.412	0.046	
Task 3	−1.025	0.441	0.020	
grey mouse lemurs: Task 2	−0.782	0.554	0.158	0.005
grey mouse lemurs: Task 3	−1.844	0.577	0.001	
persistence^a^	0.880	0.185	<0.001	<0.001
*test statistics*	*χ*² = 70.182	d.f. = 6	*p* ≤ 0.001	
**(*e*) fixed factors**				
intercept (Mme Berthe's mouse lemurs, Task 1)	2.491	0.354	< 0.001	
grey mouse lemurs	0.686	0.475	0.149	
Task 2	0.167	0.381	0.661	
Task 3	−0.213	0.401	0.596	
grey mouse lemurs: Task 2	−0.779	0.534	0.144	<0.001
grey mouse lemurs: Task 3	−2.223	0.563	< 0.001	
errors^a^	−0.335	0.138	0.016	0.017
*test statistics*	*χ*² = 48.289	d.f. = 6	*p* ≤ 0.001	
**(*f*) fixed factors**				
intercept (Mme Berthe's mouse lemurs, Task 2)	2.478	0.723	0.001	
grey mouse lemurs	0.164	0.802	0.838	
Task 3	3.150	2.258	0.163	
grey mouse lemurs: Task 3	−9.460	2.825	0.001	
change latency^a,b^	−0.042	0.672	0.950	
change latency^a,b^: grey mouse lemurs	−0.105	0.818	0.898	
change latency^a,b^: Task 3	−3.194	1.813	0.078	
change latency^a,b^: grey mouse lemurs: Task 3	7.793	2.403	0.001	<0.001
*test statistics*	*χ*² = 37.74	d.f. = 7	*p* = 0.001	

^a^*z*-transformed to a mean of 1 and a standard deviation of 0.

^b^Log-transformed.

**Figure 6.**
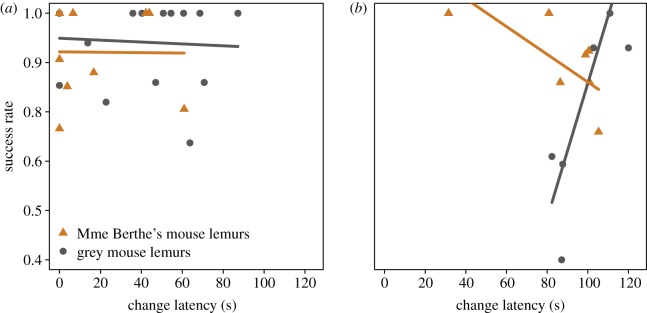
Influence of inhibitory control, measured as change latency, on the success rate for Madame Berthe's mouse lemurs (orange triangles) and grey mouse lemurs (grey circles) in Task 2 (*a*) and Task 3 (*b*). (*a*) In the second novel problem, the success rate was increased with greater inhibitory control (shorter change latency) for both species. (*b*) In the modified problem, the success rate was increased with greater inhibitory control in Madame Berthe's mouse lemurs, but with lower inhibitory control (longer change latency) in grey mouse lemurs.

Individuals with a shorter approach latency had a higher success rate (table 6*c*). Individuals that manipulated the task more persistently (table 6*d*) and which made fewer errors prior to the first success also had a higher success rate (table 6*e*). The change latency affected the success rate in interaction with species and task (table 6*f*). Madame Berthe's mouse lemurs that had a shorter change latency in both tasks had a higher success rate. Grey mouse lemurs that had a shorter change latency in Task 2 but a longer change latency in Task 3 had a higher success rate. The latter effect was the most prominent effect within the species–task interaction of the change latency affecting the success rate (figure 6).

## 4. Discussion

Using an artificial problem-solving box, we investigated whether the more generalist grey mouse lemur is more innovative than its sympatric, more specialized sister species, Madame Berthe's mouse lemur. Both species were innovative in finding a solution to a novel problem and in modifying a solution to an old problem. However, modifying a solution to an old problem appeared to be more challenging because in both species fewer individuals solved Task 3, and it also took them longer to find a solution, compared to the invention of the novel solutions in Tasks 1 and 2. Neither the personality trait exploration speed, nor body condition affected one of the performance measures. Instead, shorter approach latency, greater persistence, fewer errors prior to the first success and a generally greater inhibitory control (i.e. shorter change latency) increased performance. In contrast to our prediction, the generalist grey mouse lemurs were less successful than the ecologically more specialized Madame Berthe's mouse lemurs in modifying a solution to a familiar problem.

### 4.1. Personality and problem-solving abilities

By using standardized open field and novel object tests to assess personality [76,84], only exploration speed was repeatable over the two test series, whereas active and passive exploration as well as neophilia were not. These findings are in contrast to an earlier study of personality in grey mouse lemurs [49]. Although we used the same open field tests and comparable novel objects (plastic toy car (10 cm) or plastic duck (5 cm) [49]), these personality traits were not repeatable. This might be either due to the rather small sample size (only 17 of 54 individuals repeated both tests) or due to the location where the tests were conducted. Whereas we conducted the tests in the facilities of the research station, Dammhahn & Almeling [49] conducted the experiments directly in the forest. The forest environment might have been more familiar for mouse lemurs and may have hence influenced individuals' explorative tendencies. In our experiments, individuals that spent more time passively exploring the open field via visual and olfactory cues were more likely to approach the artificial problem-solving box, whereas neophilia did not influence familiarization with the problem-solving box. Since both traits were not repeatable, however, they may not consistently influence problem-solving abilities.

The repeatable personality trait exploration speed did not predict problem-solving performance in mouse lemurs, however. This finding is in contrast to previous studies, suggesting that faster exploring individuals were more successful problem solvers than slower explorers [3,5,30,33,90]. However, in common mynas (*Acridotheres tristis*), more explorative individuals were slower problem solvers than less explorative individuals [23], whereas in great tits (*Parus major*) more explorative individuals did not differ in problem-solving performance from less explorative individuals [91]. Differences in this relationship might be the result of differences in assessing personality traits [75]. While open field and novel object tests are common tests to measure animals’ explorative and neophilic tendencies, there is variation in how personality tests are conducted. For example, in common mynas, novel object tests were conducted directly in the familiar aviary [23], in carib grackles (*Quiscalus lugubris*), open field tests were conducted directly after capture [3], whereas in great tits open field tests were conducted in the following morning after capture [91]. Hence, the level of familiarization with the new environment may influence the outcome of these tests, which may also explain variation in personality traits between our and the previous study [49]. In addition, personality traits are sometimes not assessed by independent personality tests but by measures of behavioural variation during problem-solving tasks. For example, the latency to get in contact with the problem-solving task has been used as a proxy for neophilia or boldness or manipulatory diversity of the task as proxy for exploration (e.g. [25,92]). Thus, personality traits and problem-solving performance might be correlated *per se* as they are measured within exactly the same test.

By measuring the body condition, we estimated the effect of motivation on problem-solving performance. More specifically, motivation promoted by necessity, i.e. hunger should favour individuals in poorer body condition to innovate more [93]. For example, body condition affected problem-solving performance in a food extraction task in wild grey mouse lemurs, with individuals with lower BMI being more likely to solve the task and to do so with a better performance [69]. Alternatively, innovations could be promoted by cognitive capacity, which would then favour individuals in better body condition as they would be able to allocate available energy budgets to cognitive processes [93]. For example, pheasant chicks in better body condition entered a testing chamber earlier than pheasants in less good body condition and were subsequently more likely to participate in later sessions [94]. However, in both species of mouse lemurs, this morphology-based measure of motivation did not influence innovative abilities, which is in line with other studies [3,19,71,94,95] but in contrast to the aforementioned study on problem-solving abilities in grey mouse lemurs [69]. This study was conducted at the end of the rainy season, where mouse lemurs are generally in better body condition, and our study was conducted during the dry season where mouse lemurs are generally in a less good body condition. Hence, the individuals were equally motivated to participate in the experiments to get access to the rewards. Moreover, a potential effect of body condition might be hidden when innovations in mouse lemurs would be promoted by both necessity and cognitive capacity, which would show contrasting effects of body condition. Alternatively, cognitive capacities might rather be influenced by physiological conditions related to long-term chronic stress or concentration of antioxidants related to environmental variation [93,96], but this seems rather unlikely for our study animals, as they mostly had an age of less than a year and except for the changing seasonality, the animals did not experience any extreme environmental conditions. Possible relations of body condition and problem-solving performance therefore require further tests.

Approach latency, persistence and inhibitory control were repeatable over the three tasks, and, hence, describe personality traits. According to Sol *et al.* [92], approach latency and persistence can both represent motivation measures, which can also be considered as personality traits. Because animals were habituated to the artificial problem-solving box prior to the experiment, approach latency cannot be considered as neophobia, however [29]. Instead, considering it as motivation to engage with the task indicates that more motivated individuals achieve greater problem-solving performance than less motivated individuals, as suggested by several previous studies [18,97,98].

Additionally, repeatable persistence reflects task-directed motivation, which has been suggested to be one of the main predictors for problem-solving abilities [19] and might be driven by necessity as it has been linked to food deprivation time [9]. Yet, as in several other species [5,9,27,71], more persistent mouse lemurs achieved greater performance than less persistent individuals. In these studies, persistence was measured as time spent manipulating [5,71] or as the number of attempts to solve the problem [9,27]. As Benson-Amram & Holekamp [71] stated ‘persistence alone does not necessarily lead to greater problem-solving success' (p. 6), we tried to improve our measure of persistence by measuring persistence as the ratio of actively manipulating the box to the total time being in contact with it (active manipulation versus passive box contacts). With this ratio, we intended to measure an individual's manipulative effort as the ratio of effective behaviour to total willingness to confront the problem accounting for motivation to actively manipulate the task.

Variation in inhibitory control had an effect on whether animals solved the tasks or not and how fast they solved them. Consistent with several other studies [70,99,100], successfully innovative mouse lemurs were better able to change manipulation skills required to open the box (Task 2) and to inhibit the previously learned technique (Task 3). In addition, mouse lemurs did not need much more time to retrieve the reward after changing the respective technique, which is reflected by the positive correlation between success latency and change latency. However, the effect of inhibitory control on the success rate is less intuitive, whereas Madame Berthe's mouse lemurs exhibiting more inhibitory control were also more successful, the effect was reversed for grey mouse lemurs in Task 3.

In both species, more individuals solved the second than the first novel problem and fewer individuals solved Task 3. Since the first novel problem was the first artificial problem the animals had to solve, they needed most likely more time to develop an effective manipulation technique to solve it in comparison to the second novel problem. Here, the animals were already experienced as they had successfully manipulated the box in numerous trials during the first novel problem. This is also reflected by the higher success rate during the second novel problem compared to the first novel problem, although the former was more complex. Task 3, by contrast, represented not only the most complex task, but also a modification of the previous task. Mouse lemurs had to inhibit previously successful manipulation techniques to discover a new one. Since fewer individuals of both species were able to solve this task, only individuals exhibiting inhibitory control were able to modify a solution to an already known problem.

Mouse lemurs that made fewer errors until the first successful trial were more likely to solve the task and achieved a subsequently higher success rate. Animals either succeed because they were able to associate the manipulation technique with the food reward or as a result of trial-and-error manipulations. In both cases, if the animal could associate the manipulation technique with access to the reward, it should be likely to use this technique again, following the rules of positive reinforcement in operant conditioning [19,101]. Thus, it will achieve a greater success rate and it will be more likely to reach the learning criterion. However, if the animal is not able to associate the manipulation with access to the reward, it will proceed with any manipulation following trial-and-error, and the chance to succeed should be reduced and result in a lower success rate and a subsequently lower chance to reach the learning criterion. Furthermore, with a later first success, animals may have tried more unsuccessful manipulation techniques, which must all be inhibited in order to develop an actual successful manipulation technique. Hence, animals would be less likely to succeed in following trials, resulting in a longer success latency, a lower success rate and a lower chance to reach the learning criterion. Accordingly, an animal's lower learning ability might be due to its reduced inhibitory control, preventing animals from using other behaviours instead of the known successful one [100].

### 4.2. Interspecific problem-solving performance

Ecological generalists have been suggested to be more flexible and to better adapt to changing conditions because they are, by definition, exposed to diverse environmental conditions. Compared to specialists, they face a greater variety of problems that have to be solved in order to cope with variable habitats and food types [99]. Previous studies investigating associations with innovation rates in more than 100 North American bird species suggested that innovativeness is predicted by the degree of generalism [35,102]. Innovation rates, measured as the number of new food ingestions or the use of new foraging techniques, correlated with habitat generalism, measured as the total number of habitats used [35]. Species exhibiting a higher propensity to innovate lived in more different habitats and had a broader diet [102]. However, in our study, both mouse lemur species were able to innovate in the face of novel problems and a modified problem. Interestingly, the generalists were, on average, not more likely than the specialists to solve the tasks or to do so with a better performance, irrespective of task difficulty. In fact, the specialists even outperformed the generalists in the most complex modified problem. They also succeeded faster than the generalists and tended to be more likely to solve the tasks. Whereas the generalist and specialist species did not differ in their innovative ability to find a solution to a novel problem, the specialist species was more effective in finding a novel solution to a modified problem.

Interspecific variation in problem-solving performance could be explained by (i) personality differences, (ii) differences in foraging patterns, (iii) different energy expenditure or (iv) executive control. First, individual specialization can be the result of specific personality traits in that consistent activity, exploration or boldness determines the type or quantity of consumed food resources [103]. In primates, for example, generalist species exhibit more diverse behavioural variability than specialists [104]. Moreover, generalists appear to be more explorative and less neophobic than specialists which is, in turn, correlated with innovative abilities [39]. However, we could not find differences in personality in the two mouse lemur species, which might be due to the fact that only some personality traits were repeatable in this study.

Second, species with different diets need different skills to acquire food resources. For example, frugivorous lemurs exhibit better spatial memory and better motor control than species with a mixed diet or more folivorous species [40,105–107]. Moreover, in birds and primates, species exhibiting a greater dietary breadth appear to have better cognitive abilities than more specialized species [34,105]. The diet of Madame Berthe's mouse lemurs is completely integrated in the broader dietary spectrum of grey mouse lemurs, however, and both species are not extractive foragers [63]. As Madame Berthe's mouse lemurs spend, depending on the season, 60–90% of their feeding time on insect secretions [63,108], their better performance in the problem-solving task cannot be explained by a generally greater reliance on extractive foraging techniques.

Third, experiencing a narrower feeding niche in combination with a small body size requires efficient feeding strategies to reach energy requirements. As Madame Berthe's mouse lemurs have a lower BMI than grey mouse lemurs and, hence, a lower basic metabolic rate, efficiency might be more important to maintain their body condition compared to the larger grey mouse lemurs. However, we could not detect an influence of BMI on problem-solving performance.

Fourth, a narrower feeding niche makes it more important to exploit a specific food item once encountered [38]. Specialists might thus be specifically adept at solving a food-rewarded task, showing higher motivation to get in contact with the task, higher persistence to manipulate it and a greater ability to relearn a manipulation technique once a task changed. Inhibiting an unsuccessful technique, in general, enhances problem-solving efficiency, as inappropriate motor responses delay or prevent later success and flexibility in executive control might vary with ecology, including the level of generalism [40]. In primates, species with a greater dietary breadth exhibited superior inhibitory control [105]. Although both species were equally motivated to participate in the task, Madame Berthe's mouse lemurs were more persistent than grey mouse lemurs. Additionally, Madame Berthe's mouse lemurs exhibiting a better inhibitory control in the second novel and the modified problem were more successful. By contrast, grey mouse lemurs exhibiting a better inhibitory control were more successful only in the second novel problem, and this effect was reversed in the modified problem. Thus, the unexpected superiority of the specialists might more likely result from enhanced executive control compared to the generalists.

## 5. Summary and conclusion

In summary, we could show that in sympatric sister species of which one is an ecological generalist and the other an ecological specialist, innovators were more motivated to engage with and to manipulate a task, and that these factors also positively influenced problem-solving performance. Furthermore, an early first success promoted later successful problem-solving, as did greater motivation and inhibitory control. In addition, novel problems were solved more often than the more complex modified problem. Madame Berthe's mouse lemurs, in which cognitive abilities were examined for the first time in this study, were more innovative than grey mouse lemurs, most likely because they exhibited more inhibitory control, but not because of differences in personality traits. Hence, our results do not support the notion that generalist species are generally more innovative than specialist species and that the ability to innovate is more variable within species than between them. Hence, our study provides important new insights for the field of interspecific variation in innovativeness. It is the first to compare innovative abilities in two wild living sister species experiencing similar environmental conditions.

## Supplementary Material

Data table

## Supplementary Material

Personality data

## Data accessibility

The dataset supporting this article has been uploaded as part of the electronic supplementary material.
